# The impact of competing stroke etiologies in patients with atrial fibrillation

**DOI:** 10.1177/23969873231185220

**Published:** 2023-07-04

**Authors:** Annaelle Zietz, Alexandros A Polymeris, Fabrice Helfenstein, Sabine Schaedelin, Lisa Hert, Benjamin Wagner, David J Seiffge, Christopher Traenka, Valerian L Altersberger, Tolga Dittrich, Josefin Kaufmann, Flavia Ravanelli, Joachim Fladt, Urs Fisch, Sebastian Thilemann, Gian Marco De Marchis, Henrik Gensicke, Leo H Bonati, Mira Katan, Urs Fischer, Philippe Lyrer, Stefan T Engelter, Nils Peters

**Affiliations:** 1 Department of Neurology and Stroke Center, University Hospital Basel and University of Basel, Basel, Switzerland; 2 Neurology and Neurorehabilitation, University Department of Geriatric Medicine Felix Platter, University of Basel, Switzerland; 3 Clinical Trial Unit, Bern University, Bern, Switzerland; 4 Department of Clinical Research, University Hospital Basel and University of Basel, Switzerland; 5 Department of Intensive Care Medicine, University Hospital Basel, Switzerland; 6 Department of Neurology and Stroke Center, Inselspital, Bern, Switzerland; 7 Department of Neurology, Kantonsspital St. Gallen, St. Gallen, Switzerland; 8 Department for Neurology, University Hospital Zurich, Switzerland; 9 Stroke Center, Klinik Hirslanden, Zürich, Switzerland

**Keywords:** Stroke, stroke etiology, atrial fibrillation, large artery atherosclerosis, oral anticoagulation

## Abstract

**Background:**

Data on the impact of competing stroke etiologies in stroke patients with atrial fibrillation (AF) are scarce.

**Methods:**

We used prospectively obtained data from an observational registry (Novel-Oral-Anticoagulants-in-Ischemic-Stroke-Patients-(NOACISP)-LONGTERM) of consecutive AF-stroke patients treated with oral anticoagulants. We compared the frequency of (i) the composite outcome of recurrent ischemic stroke (IS), intracerebral hemorrhage (ICH) or all-cause death as well as (ii) recurrent IS alone among AF-stroke patients with versus without competing stroke etiologies according to the TOAST classification. We performed cox proportional hazards regression modeling adjusted for potential confounders. Furthermore, the etiology of recurrent IS was assessed.

**Results:**

Among 907 patients (median age 81, 45.6% female), 184 patients (20.3%) had competing etiologies, while 723 (79.7%) had cardioembolism as the only plausible etiology. During 1587 patient-years of follow-up, patients with additional large-artery atherosclerosis had higher rates of the composite outcome (adjusted HR [95% CI] 1.64 [1.11, 2.40], *p* = 0.017) and recurrent IS (aHR 2.96 [1.65, 5.35 ], *p* < 0.001), compared to patients with cardioembolism as the only plausible etiology. Overall 71 patients had recurrent IS (7.8%) of whom 26.7% had a different etiology than the index IS with large-artery-atherosclerosis (19.7%) being the most common non-cardioembolic cause.

**Conclusion:**

In stroke patients with AF, causes other than cardioembolism as competing etiologies were common in index or recurrent IS. Concomitant presence of large-artery-atherosclerosis seems to indicate an increased risk for recurrences suggesting that stroke preventive means might be more effective if they also address competing stroke etiologies in AF-stroke patients.

**Clinical Trial Registration:**

NCT 03826927

## Graphical abstract

**Graphical abstract:**
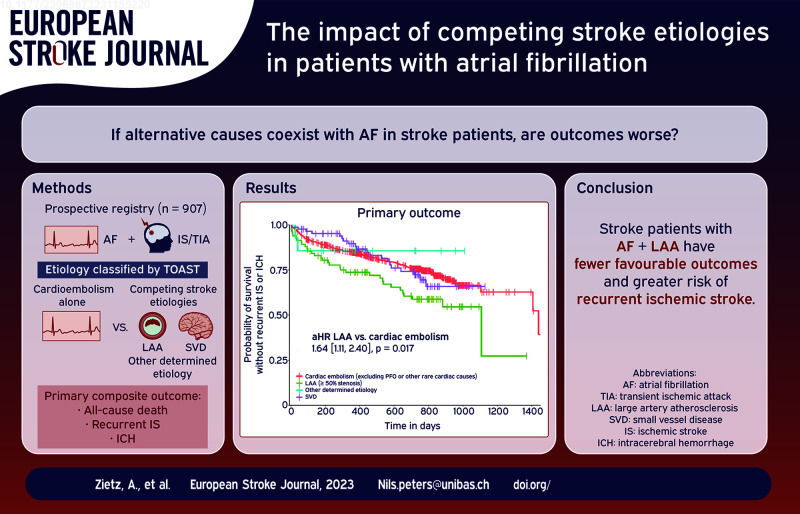

## Introduction

Cardioembolism (CE) related to atrial fibrillation (AF) is a major cause for ischemic stroke (IS). Oral anticoagulation (OAC) is the treatment of choice for primary and secondary stroke prevention in AF patients.^1,2^ Nevertheless, acute IS despite OAC in AF patients does occur in clinical practice^3,4^ challenging physicians whether and how stroke prevention strategy can be improved.

In a multicenter case control study (RENo Study),^5^ inadequate low dose of direct oral anticoagulants (DOAC), atrial enlargement, hyperlipidemia and a high CHA_2_DS_2_-VASc score were found to be associated with the recurrence of ischemic events. Here, the proportion of cardioembolic stroke was 63.9% using the ASCOD (A for atherosclerosis; S for small-vessel disease, C for cardiac pathology, and O for other causes D for dissection) classification system.^6^ Given the co-occurrence of AF, large artery atherosclerosis (LAA) and small vessel disease (SVD) in the elderly population, AF may not be the only mechanism leading to IS. Data on the prognostic impact of competing etiologies in AF patients is scarce. In addition, knowledge on the etiology of the recurrent IS in this patient cohort is limited. Further investigations on the prognostic role of distinct etiologies in IS patients with AF may be helpful to improve strategies for stroke prevention.

With these considerations in mind, we aimed to comprehensively compare AF-stroke patients with CE as the only plausible index stroke etiology to those with competing etiologies regarding the frequency of (i) a composite outcome including recurrent IS, intracranial hemorrhage (ICH) or death and (ii) recurrent IS alone during follow-up. Furthermore, we systematically assessed the etiology of recurrent IS and the difference of etiology between index and recurrent IS.

## Patients and methods

### Study design and patient cohort

This analysis is based on prospectively collected data from the registry entitled “Novel oral anticoagulants in Ischemic Stroke Patient (NOACISP)-LONGTERM” enrolling adult AF patients with acute recent (<3 months) IS (i.e. acute focal neurological deficits with a corresponding lesion and/or persistent deficit >24 h), transient ischemic attack (TIA) (i.e. acute focal neurological deficits of presumed ischemic origin lasting <24 h) or intracerebral hemorrhage (ICH) between April 2013 and December 2020 as described previously.^7,8^ All patients were treated with OAC, while the prescribed type (i.e. vitamin K antagonist (VKA) or DOAC) was chosen by consensus of patients and the treating physicians. Follow-up data were obtained by scheduled visits 3, 6, 12, and at least 24 months after inclusion. The visits were conducted by trained study personnel using standardized forms through telephone calls, out-patient visits, and hospital or general practitioner’s records until October 30th 2021.

For the current study we included patients from the registry with (i) non-valvular AF (ii) presenting with IS or TIA, (iii) presence of data about the etiology of the index and, if applicable, a recurrent IS, based on the criteria of the TOAST classification^9^ and a follow-up period of at least 3 months after the index event. We excluded patients with ICH as index event.

In line with previous studies^7,8^ we used the following variables from NOACISP-LONGTERM: age, sex, body mass index, hypertension, diabetes, hyperlipidemia, peripheral artery disease, regular alcohol consumption, active smoking, history of IS and/or ICH, and the CHA_2_DS_2_-VASc score,^1^ the initial clinical presentation using the National Institutes of Health Stroke Scale (NIHSS)^10^ as well as the antithrombotic therapy before and after the index event and concomitant medications. Follow-up data up included (i) functional outcome measured by the modified Rankin scale (mRS)^11^ (ii) occurrence of recurrent IS with the etiology based on the TOAST classification, (iii) ICH, or (iv) all-cause death during follow up.

The study followed the STROBE guidelines.^12^

### Study outcomes

Our primary outcome was the time between index event to the composite of recurrent IS, ICH, and all-cause death. Our secondary outcome was recurrent IS alone.

### Competing stroke etiologies

The index event was classified using the TOAST classification by the study physician that included the patient in the registry. We operationalized the presence of competing stroke etiologies by applying the TOAST classification as follows. For patients with *two or more potential causes of stroke* the study physician named the potential competing stroke etiologies (e.g. cardioembolism (CE) and large artery atherosclerosis (LAA) or small vessel disease (SVD) and CE). Taking into consideration that every patient had atrial fibrillation and thus CE generally as a potential etiology, we regrouped the patients as follows (i) **CE only** were patients with cardioembolism as the sole plausible etiology. (ii) **LAA+**: included patients with LAA (defined as stenosis greater than >50% of an appropriate intracranial or extracranial artery) as most likely etiology or as potential competing stroke etiology identified in *two or more potential causes of stroke* (iii) **SVD+**: included patients with SVD as either most likely etiology or as competing stroke etiology identified in *two or more potential causes of stroke*. (iv) **Other determined etiology**: including patients with other determined etiology as suspected main etiology or as potential competing stroke etiology identified in *two or more potential causes of stroke*.

### Statistical analysis

We compared patients using descriptive statistics stratified by the occurrence of the primary or secondary outcome during follow-up. We used the Chi^2^ test to compare the categorical variables, we presented the data accordingly with numbers and proportions. For continuous variables we used the *t*-test and reported the mean values and standard deviations. In case of non-normally distributed data, the Whitney *U* test or the Kruskal-Wallis rank sum test was used.

We performed time-to-event-analyses for the primary and secondary outcomes in relation to the index stroke etiology using cox proportional hazard models with Firth penalization for rare events if necessary. The follow-up time was censored at the last visit or death for patients who had no outcome event. We adjusted the models for the following known confounding factors age, sex, hyperlipidemia, diabetes mellitus, hypertension, smoking, concomitant statin use at admission as well as the CHA_2_DS_2_-VASc score. Only patients with complete overall data were included in the time to event analysis.

For our Kaplan-Meier curves presenting the primary and secondary outcome, curves were stratified by the index stroke etiology with CE as the reference level.

We performed a post-hoc sensitivity analysis excluding patients with revascularization therapy (i.e. carotid endarterectomy (CEA) or carotid stenting (CAS)) with LAA+ as competing stroke etiology at the index event.

Statistical analyses were performed using R version 4.1.2.

## Results

Among 1060 patients of the NOACISP registry, 907 patients (85.6%) were eligible for our analyses (Supplemental Figure 1). The remaining patients were excluded for the following reasons: 31 patients with missing follow-up visits, 35 patients without definite diagnosis of AF, 3 patients with incomplete follow-up data as well as 35 patients with ICH as index event. Finally, 48 patients with stroke mimics or missing information on the index event were excluded and 1 patient with missing data on the TOAST classification of the index event.

The median age was 81 years (Interquartile range (IQR) [74, 86]), 54.4% were male and patients had a median NIHSS of 4 [2, 9] (Supplemental Table 1). One hundred and eighty-four of 907 patients (20.3%) had competing etiologies, while 723 (79.7%) had CE as sole plausible etiology of the index event.

### Primary outcome

The composite outcome occurred in 230/907 (25.3%) patients of whom 71 (31%) had an IS, 14 (6%) suffered from an ICH and 145 patients died (63%) during a median follow-up time of 2.01 years (1587 patient-years).

Patients with the occurrence of the primary composite outcome during follow-up compared to those without were older and had more often risk factors for a recurrent IS and ICH with a higher burden of concomitant vascular diseases including arterial hypertension, coronary heart disease, and peripheral artery disease. Patients with IS, ICH, or death during follow up also had higher CHA_2_DS_2_-VASc and HAS-BLED scores at baseline (see Table 1).

**Table 1.** Comparison of baseline demographics and clinical characteristics, concomitant medication and clinical information’s at baseline (index stroke) between patient who suffered from at least one primary event (i.e. recurrent acute IS, ICH, or death) and those who did not.

	No primary outcome	⩾1 primary outcome	*p*-Value
Demographics			
*N*	677	230	
Age, years (median, [IQR])	80 [74, 85]	84 [77,88]	<0.001
Male sex, *n* (%)	360 (53.2)	133 (57.8)	0.25
Medication before index event, *n* (%)			
DOAC	163 (24.1)	62 (27)	0.43
VKA	117 (17.3)	63 (27.4)	0.001
Antiplatelet	183 (27)	73 (31.7)	0.20
DOAC/antiplatelet	14 (2.1)	7 (3.0)	0.55
VKA/antiplatelet	11 (1.6)	7 (3.0)	0.29
Dual antiplatelet	3 (0.4)	0 (0)	0.73
Vascular risk factors, *n* (%)			
Hypertension	540 (79.8)	202 (87.8)	0.008
Diabetes	153 (22.6)	66 (28.7)	0.07
Hyperlipidemia	348 (51.4)	111 (48.3)	0.45
Non- Smoking	496 (73.3)	179 (77.8)	0.32
No regular alcohol consumption	517 (76.4)	175 (76.1)	0.98
Concomitant diseases, *n* (%)			
Coronary heart disease	169 (25)	82 (35.7)	0.002
Heart failure	107 (15.8)	41 (17.8)	0.54
Peripheral artery disease	53 (7.8)	35 (15.2)	0.002
Renal insufficiency	31 (4.6)	18 (7.8)	0.09
CHA2DS2-VASc-score	6 [5,6]	6 [5,7]	<0.001
HAS-BLED score	2 [2,3]	3 [2,3]	<0.001
NIHSS at index stroke	4 [2,8]	4 [2,9]	0.015
Creatinin (µmol/l), median [IQR]	83 [70, 103]	90 [72.7, 115.2]	0.002

SD: standard deviation; IQR: interquartile range; DOAC: direct oral anticoagulants; VKA: vitamin K antagonist.

LAA+ as competing stroke etiology was nearly twice as frequent (13.9% vs 7.5%) in patients with the occurrence of the primary outcome during follow-up compared to those without and slightly less patients had an index stroke due to CE alone 76.5% versus 80.8% (see Table 2).

**Table 2.** Comparison of the most likely cause of index event according to the criteria of the TOAST classification between patients who endured at least one primary outcome event (recurrent IS, ICH, or death). Categories are presented with numbers and percentages.

	No primary outcome (*n* = 677) (%)	⩾1 primary outcome (*n* = 230) (%)
Cardiac embolism (CE) only	547 (80.8)	176 (76.5)
Large artery atherosclerosis (LAA+)	51 (7.5)	32 (13.9)
Small vessel disease (SVD+)	67 (9.9)	20 (8.7)
Other determined etiology	12 (1.8%)	2 (0.9)

**Figure 1.**
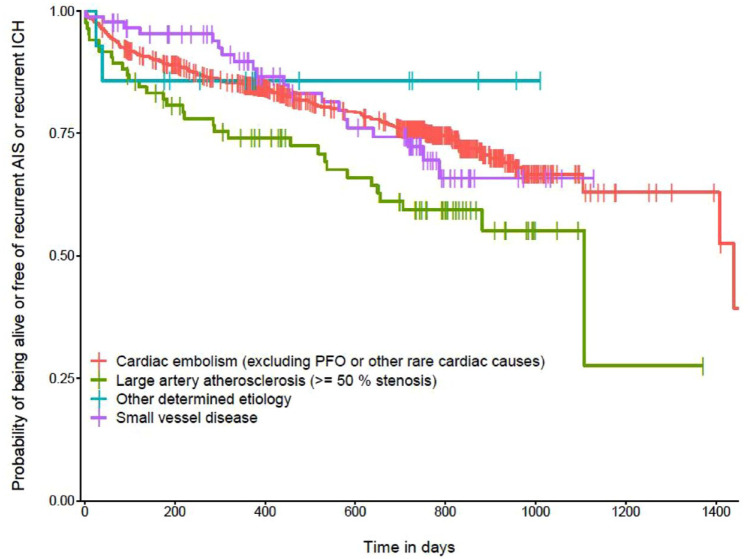
Kaplan-Meier curve representing the probability of being alive or free of the composite outcome compromising recurrent ischemic stroke (IS), intracerebral hemorrhage (ICH) and death in relation to the potential index stroke etiology based on the TOAST classification. The reference level of the interpretation of hazard ratios is cardiac embolism. Vertical dashes represent censored data. Analysis were adjusted for age, sex, hyperlipidemia, diabetes mellitus, hypertension, the CHA_2_DS_2_-VASc score, smoking and statin at admission. One patient was excluded due to missing data.

An index event with LAA+ as competing stroke etiology was associated with the primary composite outcome after adjusting for age, sex, hypertension, diabetes, hyperlipidemia, the CHA_2_DS_2_-VASc score, smoking, and concomitant statin use at admission (aHR 1.64, 95% CI [1.11, 2.40], *p* = 0.017, Figure 1) such an association was absent in SVD+ (aHR 0.85, 95% [0.53, 1.36], *p* = 0.49, Figure 1).

### Secondary outcome

The baseline characteristics of patients with recurrent IS compared to patients without were balanced; however, the subgroups differed with respect to the presence of peripheral artery disease (PAD), hyperlipidemia, and the intake of direct oral anticoagulants before the index event (see Supplemental Table 2).

In Table 3 the stroke etiologies according to the occurrence of the secondary outcome are demonstrated. In patients with the occurrence of a recurrent IS during follow-up compared to those without recurrent IS, the proportion of an index event due to CE alone was lower (47/71 patients (66.2%) vs 676/836 patients (80.8%)) and LAA+ was more frequent as competing index stroke etiology (16/71 patients (22.5%) vs 67/836 patients (8%)).

**Table 3.** Comparison of the potential cause of index event according to the TOAST classification between patients with and without at least one recurrent IS during follow up. In three patients the secondary outcome was missing. Categories are presented with numbers and percentages.

	No recurrent acute IS (*n* = 836) (%)	⩾1 recurrent acute IS (*n* = 71) (%)
Cardiac embolism (CE) only	676 (80.8)	47 (66.2)
Large artery atherosclerosis (LAA+)	67 (8)	16 (22.5)
Small vessel disease (SVD+)	80 (9.6)	7 (9.9)
Other determined etiology	13 (1.6)	1 (1.4)

**Figure 2.**
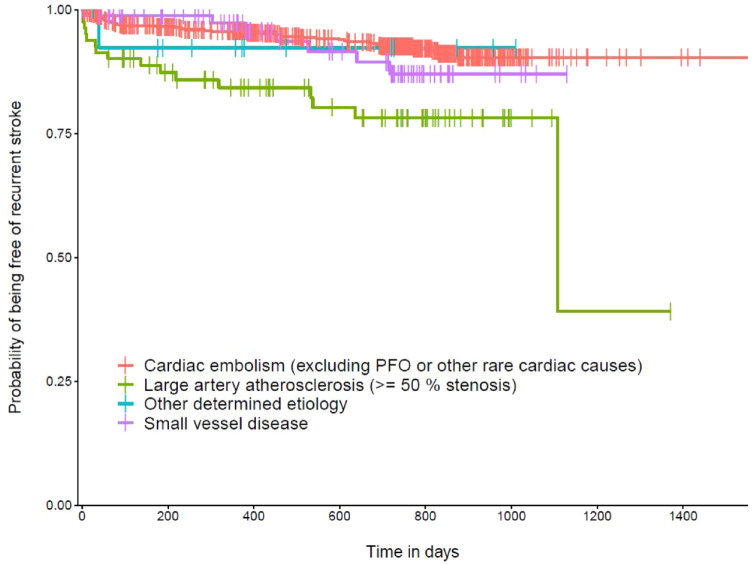
Kaplan-Meier curve representing the probability of being free a recurrent ischemic stroke (IS) in relation to the potential index stroke etiology based on the TOAST classification. The reference level of the interpretation of hazard ratios is cardiac embolism. Vertical dashes represent censored data. Analysis were adjusted for age, sex, hyperlipidemia, diabetes mellitus, hypertension, the CHA_2_DS_2_-VASc score smoking and statin at admission. Thirty-two patients were excluded due to missing data.

In our adjusted time to event analysis, LAA+ (aHR 2.96, 95% CI [1.65, 5.35], *p* < 0.001) was associated with a higher risk for a recurrent IS compared to patients with an index event due to CE alone as illustrated in Figure 2. No association was found for SVD+ (aHR 1.36, 95% CI [0.60–3.04], *p* = 0.465).

### Post-hoc sensitivity analysis

Additionally, we performed a post-hoc sensitivity analysis excluding 17 patients (CEA: *n* = 13, CAS: *n* = 4) with revascularization therapy out of 83 patients with LAA+ as competing index etiology.

Regarding our primary outcome, the hazard ratio was elevated in subjects with LAA+ as competing etiology compared to CE alone although missing statistical significance (aHR 1.46 [0.95, 2.26], *p* = 0.095, also see Table 4 in the Supplemental Material). LAA+ remained significantly associated with recurrent ischemic stroke (aHR 2.57 [1.31, 5.04], *p* = 0.011, also see Table 5 in the Supplemental Material).

### Etiology of the recurrent IS

In 70 of 71 patients (98,6%) with recurrent IS, the TOAST classification was available. In 46/71 (64.8%) patients with recurrent IS the etiology was CE alone, LAA+ in 14/71 patients (19.7%), SVD+ in 8/71 (11.3%) and other determined etiology in 2/71 (2.8%), respectively (also see table 4).

**Table 4.** Etiology of the recurrent IS according to the TOAST classification.

Etiology of the recurrent IS (*n* = 71)	Cases (%)
Cardiac embolism (CE) only	46 (64.8)
Large artery atherosclerosis (LAA+)	14 (19.7)
Small vessel disease (SVD+)	8 (11.3)
Other determined etiology+	2 (2.8)
Missing data	1 (1.4)

Categories are presented with number and percentages.

**Figure 3.**
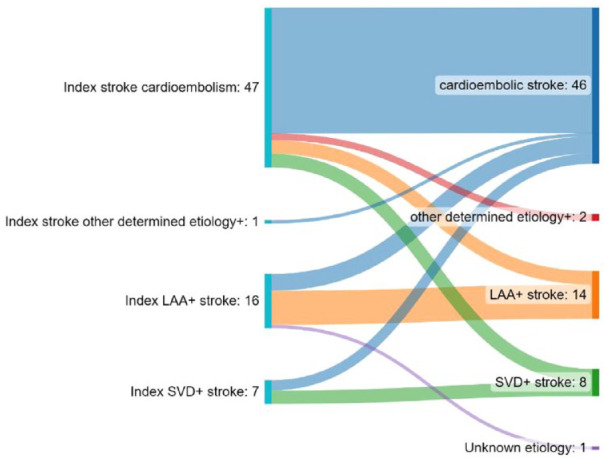
Detailed Illustration of patients with recurrent ischemic stroke. On the left side, the index stroke etiology is noted, on the right side the etiology of the recurrent stroke based on the TOAST classification. The flows represent the changes between the index and recurrent stroke etiology. TOAST classification is missing for one recurrent event (*n* = 71).

As illustrated in Figure 3, in patients with recurrent IS the etiologies differed between the index and recurrent event in 19/71 (26.7%) of the cases.

## Discussion

This registry-based study on the prognostic impact of competing stroke etiologies among AF-stroke patients had the following key finding: (i) competing stroke etiologies were present in about every fifth AF-stroke patient. (ii) LAA+ indicated a higher risk for complications including recurrent IS in comparison to patients with CE as the only plausible etiology of the index event, including patients that underwent revascularization treatment for carotid artery stenosis. (iii) Index stroke and recurrent stroke differed regarding their etiology in more than a quarter of the patients and LAA+ was the most important non-cardioembolic etiology.

The frequency of non-cardioembolic stroke at the index event in AF patients was described in other studies – where LAA was reported in 11.3% and SVD in 13.2%^13^ – recording similar rates compared to our study (20.3%).

Recent studies on AF patients focused on the impact of competing etiologies in acute IS despite OAC, a patient population with a high risk of recurrent events.^14^ Competing stroke etiologies were reported in 24% by Polymeris et al.,^3^ while other studies described slightly higher rates (32.7%) in stroke patients under OAC^5^ based on the ASCOD classification.^6^

We demonstrated that LAA+ as competing stroke etiology was significantly associated with the primary composite endpoint in a stroke population with AF. Interestingly, in a prior study investigating competing stroke etiologies, patients with CE and LAA as potential stroke etiology had the highest rate of all-cause mortality following the index event compared to other stroke etiologies, pointing toward an important high risk patient population,^15^ although data on IS and ICH as clinically important outcome events were lacking.

Looking at recurrent IS alone, LAA+ as competing stroke etiology at the index event was associated with an elevated risk for recurrent IS compared to CE as only plausible index etiology. Taking into consideration that LAA is known to be associated with a high risk for recurrent IS in a general stroke population^16^ our findings focused on patients with AF-related stroke underlines the poor prognostic meaning of concomitant LAA in AF patients. This is clinically relevant because concomitant LAA in AF patients is common, in elderly patients percentages up to 12% were reported.^17^

In our study, an index event due to SVD was not associated with our primary or secondary outcome. However, we did not incorporate imaging markers of SVD that has been shown to be associated with a higher risk for recurrent IS in stroke patients with AF.^18^ Even though neuroimaging marker of SVD were associated with recurrent events in the study of Du et al, the etiology of the recurrent IS was only attributed to SVD in 5.7%, whereas in our analysis 11.3% of the recurrent IS had SVD as potential etiology.

A recently published study demonstrated that competing stroke etiologies were more common in AF stroke patients despite OAC compared to those without prior OAC. In particular, small vessel occlusion and arterial atheroma were associated with IS despite OAC.^19^ In contrast to this cross sectional study, we were able to investigate the occurrence of recurrent events (including the etiology of recurrent IS) in detail, demonstrating that especially LAA+ at the index event was associated with recurrent IS. Targeting competing stroke etiologies in AF patients at the index event may thus be relevant to reduce the risk for a recurrent IS under OAC.

Of note, 17/83 patients with LAA+ as competing stroke etiology received a revascularization therapy. A large part of the LAA+ patients (79.5%) received no carotid endarterectomy (CEA) or a carotid stenting (CAS). Treatment decision was based on interdisciplinary neurovascular board recommendation. Potentially the rather low rate of performed revascularization treatment in these patients with both, LAA and AF, may point toward a less stringent indication for revascularization in stroke patients with AF and competing etiologies. Overall, our findings of an increased risk for both the primary and secondary outcome in the presence of LAA were observed for the overall cohort, including subjects that underwent revascularization. Especially the risk of recurrent IS was consistent, irrespective of the inclusion or exclusion of patients that received an interventional treatment of the carotid stenosis, which may also reflect the general high vascular burden in this at risk population.

The etiologies of the index and recurrent IS varied in approximately one quarter of the cases in our study. These findings underline that an effective secondary stroke prevention strategy based on the etiology of the index event does not necessarily prevent a recurrent event due to a different etiology. Thus, the treatment of shared cardiovascular risk factors and potential causes of concomitant diseases such as SVD and LAA should not be overlooked in stroke patients with AF.

Up to date, no clear benefit of add-on antiplatelet therapy in stroke patients with AF under OAC could by demonstrated in observational studies.^3,20^ Interestingly, a recent study demonstrated that in patients with ischemic stroke despite OAC concomitant antiplatelet use was associated with an increased risk of all-cause death, ICH and IS as well as recurrent IS alone.^3^ Data derived from cardiac studies on newly diagnosed AF patients also found a higher risk of bleeding events and IS during follow-up in patients treated with concomitant antiplatelet agents compared to OAC alone.^21^ Of note, the duration of the concomitant antiplatelet use was unknown in these observational studies and the elevated risk of recurrent ischemic events may only reflect a high risk population group with an increased vascular load than a causative effect of the antiplatelet agents. In fact, especially stroke patients with large artery arteriosclerosis are at high risk of early recurrence^16^ and short term antiplatelet use may be beneficial in this time period. However – to the best of our knowledge – no randomized controlled studies investigated the short term use of an additional antiplatelet therapy in AF-stroke patients with concomitant LAA. Our findings underline that AF associated stroke patients witch competing stroke etiologies – majorly concomitant LAA – have a substantial residual risk of recurrence and further research is needed to improve secondary stroke prevention strategies.

Our study has the following limitations: (i) we used observational data and thus unknown confounders may have introduced bias even though we adjusted our analysis for variables known to affect the risk of ischemic or hemorrhagic complications. (ii) We did not perform an interrater-reliability assessment of the determination of stroke etiology and the operationalization of our dichotomization in presence versus absence of competing etiologies. However, even though the TOAST classification was not determined centrally, the stroke assessment was conducted by an experienced neurovascular study physician. (iii) The management to optimize the vascular risk factor profile including diabetes mellitus, hypertension, lipids was not standardized and details were not systematically available. (iv) The study is based on a single-center registry which reduces the generalizability of the results even though we included a rather large cohort in this analysis, treated at a specialized comprehensive stroke center, thus reflecting the current standard of care for stroke patients.

Our study has several strengths: (i) it is based on a well curated registry including AF patients on OAC following recent IS treated at a specialized stroke center, (ii) we were able to describe not only the etiology of the index event but also the recurrent IS and thus to extend and refine the findings of previous studies,^3,22^ and (iii) our results were robust also after adjustment for known vascular risk factors.

In conclusion, in stroke patients with AF stroke etiologies other than CE are common in index or recurrent IS. In particular, the concomitant presence of LAA seems to indicate an increased risk for recurrences. These observations suggest that stroke preventive means might be more effective if they also address stroke etiologies other than CE in AF-stroke patients. In particular, stroke patients with AF and concomitant LAA may be in need of a more individualized approach in secondary stroke prevention strategies.

## Supplemental Material

sj-docx-1-eso-10.1177_23969873231185220 – Supplemental material for The impact of competing stroke etiologies in patients with atrial fibrillation Click here for additional data file. Supplemental material, sj-docx-1-eso-10.1177_23969873231185220 for The impact of competing stroke etiologies in patients with atrial fibrillation by Annaelle Zietz, Alexandros A Polymeris, Fabrice Helfenstein, Sabine Schaedelin, Lisa Hert, Benjamin Wagner, David J Seiffge, Christopher Traenka, Valerian L Altersberger, Tolga Dittrich, Josefin Kaufmann, Flavia Ravanelli, Joachim Fladt, Urs Fisch, Sebastian Thilemann, Gian Marco De Marchis, Henrik Gensicke, Leo H Bonati, Mira Katan, Urs Fischer, Philippe Lyrer, Stefan T Engelter and Nils Peters in European Stroke Journal
