# Supplementary material for: The impact of competing stroke etiologies in patients with atrial fibrillation
Source: Eur Stroke J. 2023 Jul 4;8(3):703–11. doi: 10.1177/23969873231185220 (PMC10472960; doi:10.1177/23969873231185220)
Supplement: sj-docx-1-eso-10.1177_23969873231185220 – Supplemental material for The impact of competing stroke etiologies in patients with atrial fibrillation [file sj-docx-1-eso-10.1177_23969873231185220.docx]

1. **Supplementary Material**

**Supplementary Tables**

**1.1 Table 1:** Baseline characteristics of the study population

|  | Overall | Ischemic stroke | Transient ischemic attack (TIA) |
| --- | --- | --- | --- |
| Demographics |  |  |  |
| N | 907 | 832 | 75 |
| Age, years (median, [IQR]) | 81 [74, 86] | 81 [74, 86] | 81 [74, 85.5] |
| Male sex, n ( %) | 493 (54.4) | 443 (53.2) | 50 (66.7) |
| Medication before index event, n (%) |  |  |  |
| DOAC | 225 (24.8) | 205 (24.6) | 20 (26.7) |
| VKA | 180 (19.8) | 163 (19.6) | 17 (22.7) |
| Antiplatelet | 256 (28.2) | 228 (27.4) | 28 (37.3) |
| DOAC/antiplatelet | 21 (2.3) | 17 (2) | 4 (5.3) |
| VKA/antiplatelet | 18 (2) | 14 (1.7) | 4 (5.3) |
| Dual antiplatelet | 3 (0.3) | 3 (0.4) | 0 (0) |
| Vascular risk factors, n ( %) |  |  |  |
| Hypertension | 742 (81.8) | 681 (81.9) | 61 (81.3) |
| Diabetes | 219 (24.1) | 198 (23.8) | 21 (28) |
| Hyperlipidemia | 459 (50.6) | 424 (51) | 35 (46.7) |
| Non- Smoking | 675 (74.5) | 622 (74.8) | 53 (70.7) |
| No regular alcohol consumption | 692 (76.3) | 639 (76.8) | 53 (70.7) |
| Concomitant diseases n (%) |  |  |  |
| Coronary heart disease | 251 (27.7) | 228 (27.4) | 23 (30.7) |
| Heart failure | 148 (16.3) | 136 (16.3) | 12 (16) |
| Peripheral artery disease | 88 (9.7) | 81 (9.7) | 7 (9.3) |
| Renal insufficiency | 49 (5.4) | 47 (5.6) | 2 (2.7) |
| NIHSS at index stroke | 4 [2,9] | 4 [2,9] | 1 [0,2] |
| Creatinin (µmol/l), median [IQR] | 85 [70, 106] | 85 [70, 106] | 86 [74, 107] |
| CHA2DS2-VASc-score | 6 [5,6] | 6 [5,6] | 6 [5,6] |

* SD = standard deviation, IQR = interquartile range, DOAC = direct oral anticoagulants, VKA = vitamin K antagonist, mRS = modified Rankin Scale. Normally distributed variables are presented with mean and (SD), non-normally distributed variables are presented with median and [Inter-Quartile Range]

**1.2 Table 2:**

Comparison of baseline demographics and clinical characteristics, concomitant medication and clinical information’s at baseline (index event) between patient who suffered at least from one recurrent IS and those who did not.

|  | No recurrent acute IS (n=836) | ≥1 recurrent acute IS (n=71) | p-value |
| --- | --- | --- | --- |
|  |  |  |  |
|  |  |  |  |
| Age, years (median, [IQR]) | 80.5 [74, 86] | 81 [74,86] | 0.81 |
| Male sex, n ( %) | 450 (53.8) | 43 (60.6) | 0.33 |
| Medication before index event, n (%) |  |  |  |
| DOAC | 193 (23.1) | 32 (45.1) | < 0.001 |
| VKA | 162 (19.4) | 18 (25.4) | 0.29 |
| Antiplatelet | 238 (28.5) | 18 (25.4) | 0.67 |
| DOAC/antiplatelet | 18 (2.2) | 3 (4.2) | 0.48 |
| VKA/antiplatelet | 16 (1.9) | 2 (2.8) | 0.94 |
| Dual antiplatelet | 3 (0.4) | 0 (0) | 1.00 |
| Vascular risk factors, n ( %) |  |  |  |
| Hypertension | 681 (81.5) | 61 (85.9) | 0.44 |
| Diabetes | 203 (24.3) | 16 (22.5) | 0.85 |
| Hyperlipidemia | 414 (49.5) | 45 (63.4) | 0.03 |
| Non- Smoking | 620 (74.2) | 55 (77.5) | 0.31 |
| No regular alcohol consumption | 635 (76) | 57 (80.3) | 0.26 |
| Concomitant diseases n (%) |  |  |  |
| Coronary heart disease | 230 (27.5) | 21 (29.6) | 0.81 |
| Heart failure | 138 (16.5) | 10 (14.1) | 0.72 |
| Peripheral artery disease | 74 (8.9) | 14 (19.7) | 0.006 |
| Renal insufficiency | 47 (5.6) | 2 (2.8) | 0.46 |
| CHA2DS2-VASc-score | 6 [5,6] | 6 [5,6] | 0.89 |
| HAS-BLED score | 2 [2,3] | 3 [2,3] | 0.27 |
| NIHSS | 4 [2,9] | 4 [2,7.5] | 0.74 |

* SD = standard deviation, IQR = interquartile range, DOAC = direct oral anticoagulants, VKA = vitamin K antagonist,. Normally distributed variables are presented with mean and (SD), non-normally distributed variables are presented with median and [Inter-Quartile Range]

**1.3 Table 3:**

Comparison of baseline demographics and clinical characteristics, concomitant medication and clinical information’s at baseline (index event) between patient who received a revascularization therapy with carotid stenting or carotid endarterectomy and those who did not.

|  | Not revasculated (n=890) | revasculated (n=17) |
| --- | --- | --- |
|  |  |  |
|  |  |  |
| Age, years (median, [IQR]) | 81 [74, 86] | 76 [73,82] |
| Male sex, n ( %) | 479 (53.8) | 14 (82.4) |
| Medication before index event, n (%) |  |  |
| DOAC | 216 (24.3) | 9 (52.9) |
| VKA | 177 (19.9) | 3 (17.6) |
| Antiplatelet | 252 (28.3) | 4 (23.5) |
| DOAC/antiplatelet | 21 (2.4) | 0 (0) |
| VKA/antiplatelet | 17 (1.9) | 1 (5.9) |
| Dual antiplatelet | 3 (0.3) | 0 (0) |
| Vascular risk factors, n ( %) |  |  |
| Hypertension | 725 (81.5) | 17 (100) |
| Diabetes | 215 (24.2) | 4 (23.5) |
| Hyperlipidemia | 448 (50.3) | 11 (64.7) |
| Non- Smoking | 667 (74.9) | 8 (47.1) |
| No regular alcohol consumption | 680 (76.4) | 12 (70.6) |
| Concomitant diseases n (%) |  |  |
| Coronary heart disease | 246 (27.6) | 5 (29.4) |
| Heart failure | 148 (16.6) | 0 (0) |
| Peripheral artery disease | 80 (9) | 8 (47.1) |
| Renal insufficiency | 49 (5.5) | 0 (0) |
| CHA2DS2-VASc-score | 6 [5,6] | 6 [4,6] |
| NIHSS | 4 [2,9] | 3 [2,6] |

* SD = standard deviation, IQR = interquartile range, DOAC = direct oral anticoagulants, VKA = vitamin K antagonist,. Normally distributed variables are presented with mean and (SD), non-normally distributed variables are presented with median and [Inter-Quartile Range]

1. **Supplementary Figure**

**2.1 Figure 1: Study Flow Chart**


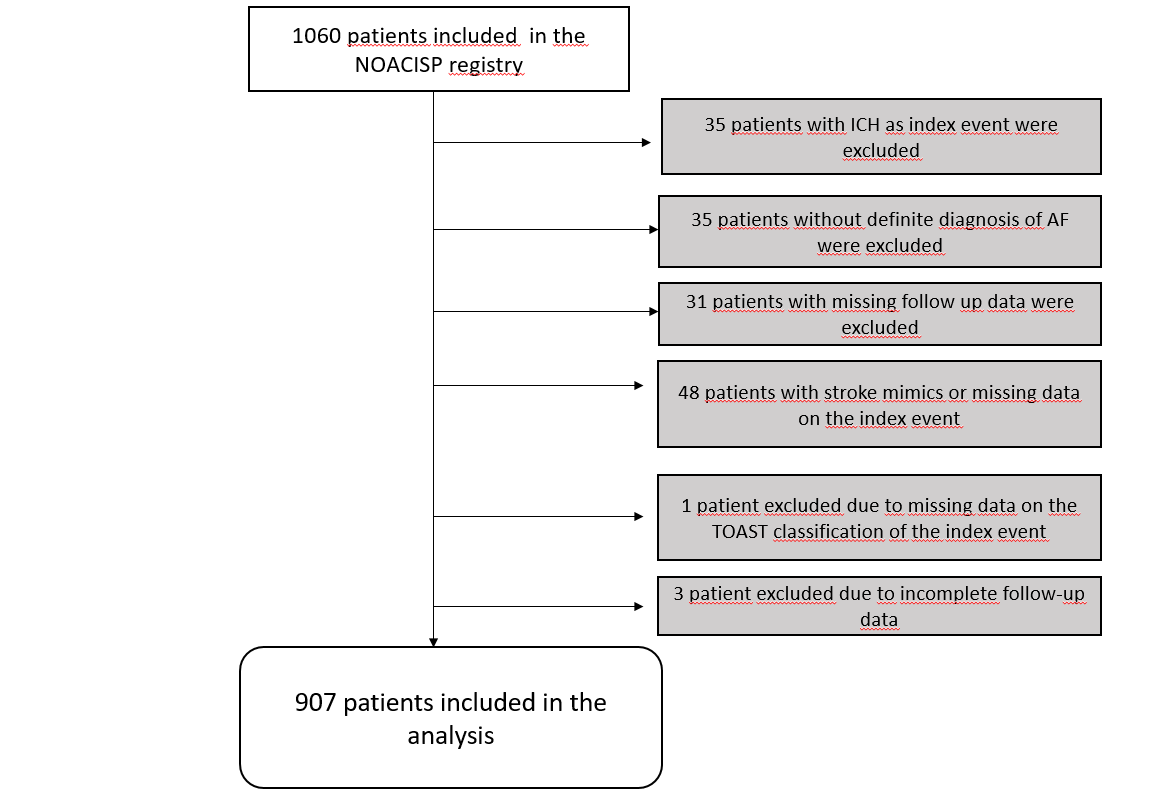


**3.Post-hoc sensitivity analysis**

**3.1 table 4**

Post-hoc sensitivity analysis for the time to event analysis regarding the primary outcome. In this Cox regression model we excluded patients in the LAA+ group with a revascularization therapy.

| **Cox regression model**  **(n=889)** | **TOAST classification of the index event** | **Adjusted estimates*** | |
| --- | --- | --- | --- |
|  |  | **Hazard ratio [95%-CI]** | **p-value** |
|  | Cardioembolism (CE) | (reference) | |
|  | Large-artery-atherosclerosis (LAA+) | 1.46 [0.95, 2.26] | 0.095 |
|  | Small vessel disease (SVD+) | 0.84 [0.53, 1.34] | 0.452 |
|  | Other determined etiology+ | 0.99 [0.28, 3.48] | 0.983 |

***** Analysis were adjusted for age, sex, hyperlipidemia, diabetes mellitus, hypertension, the CHA_2_DS_2_-VASc score.

**3.2 table 5**

Post-hoc sensitivity analysis for the time to event analysis regarding the secondary outcome. In this Cox regression model we excluded patients in the LAA+ group with a revascularization therapy.

| **Cox regression model**  **(n=858)** | **TOAST classification of the index event** | **Adjusted estimates*** | |
| --- | --- | --- | --- |
|  |  | **Hazard ratio [95%-CI]** | **p-value** |
|  | Cardioembolism (CE) | (reference) | |
|  | Large-artery-atherosclerosis (LAA+) | 2.57 [1.31, 5.04] | 0.011 |
|  | Small vessel disease (SVD+) | 1.27 [0.57, 2.84] | 0.560 |
|  | Other determined etiology+ | 1.70 [0.32, 9.03] | 0.554 |

***** Analysis were adjusted for age, sex, hyperlipidemia, diabetes mellitus, hypertension, the CHA_2_DS_2_-VASc score.
